# Nutritional Implications of Baby-Led Weaning and Baby Food Pouches as Novel Methods of Infant Feeding: Protocol for an Observational Study

**DOI:** 10.2196/29048

**Published:** 2021-04-21

**Authors:** Rachael W Taylor, Cathryn A Conlon, Kathryn L Beck, Pamela R von Hurst, Lisa A Te Morenga, Lisa Daniels, Jill J Haszard, Alison M Meldrum, Neve H McLean, Alice M Cox, Lesieli Tukuafu, Maria Casale, Kimberley J Brown, Emily A Jones, Ioanna Katiforis, Madeleine Rowan, Jenny McArthur, Elizabeth A Fleming, Ben J Wheeler, Lisa A Houghton, Aly Diana, Anne-Louise M Heath

**Affiliations:** 1 Department of Medicine University of Otago Dunedin New Zealand; 2 School of Sport, Exercise and Nutrition College of Health Massey University Auckland New Zealand; 3 Research Centre for Hauora and Health Massey University Wellington New Zealand; 4 Biostatistics Centre University of Otago Dunedin New Zealand; 5 Faculty of Dentistry University of Otago Dunedin New Zealand; 6 Department of Human Nutrition University of Otago Dunedin New Zealand; 7 Department of Women's and Children's Health University of Otago Dunedin New Zealand; 8 Nutrition Working Group, Faculty of Medicine Universitas Padjadjaran Bandung Indonesia

**Keywords:** infant, diet, complementary feeding, food pouch, baby-led weaning, iron, growth, eating behavior, feeding behavior, dental health, choking, breast milk

## Abstract

**Background:**

The complementary feeding period is a time of unparalleled dietary change for every human, during which the diet changes from one that is 100% milk to one that resembles the usual diet of the wider family in less than a year. Despite this major dietary shift, we know relatively little about food and nutrient intake in infants worldwide and virtually nothing about the impact of baby food “pouches” and “baby-led weaning” (BLW), which are infant feeding approaches that are becoming increasingly popular. Pouches are squeezable containers with a plastic spout that have great appeal for parents, as evidenced by their extraordinary market share worldwide. BLW is an alternative approach to introducing solids that promotes infant self-feeding of whole foods rather than being fed purées, and is popular and widely advocated on social media. The nutritional and health impacts of these novel methods of infant feeding have not yet been determined.

**Objective:**

The aim of the First Foods New Zealand study is to determine the iron status, growth, food and nutrient intakes, breast milk intake, eating and feeding behaviors, dental health, oral motor skills, and choking risk of New Zealand infants in general and those who are using pouches or BLW compared with those who are not.

**Methods:**

Dietary intake (two 24-hour recalls supplemented with food photographs), iron status (hemoglobin, plasma ferritin, and soluble transferrin receptor), weight status (BMI), food pouch use and extent of BLW (questionnaire), breast milk intake (deuterium oxide “dose-to-mother” technique), eating and feeding behaviors (questionnaires and video recording of an evening meal), dental health (photographs of upper and lower teeth for counting of caries and developmental defects of enamel), oral motor skills (questionnaires), and choking risk (questionnaire) will be assessed in 625 infants aged 7.0 to 9.9 months. Propensity score matching will be used to address bias caused by differences in demographics between groups so that the results more closely represent a potential causal effect.

**Results:**

This observational study has full ethical approval from the Health and Disability Ethics Committees New Zealand (19/STH/151) and was funded in May 2019 by the Health Research Council (HRC) of New Zealand (grant 19/172). Data collection commenced in July 2020, and the first results are expected to be submitted for publication in 2022.

**Conclusions:**

This large study will provide much needed data on the implications for nutritional intake and health with the use of baby food pouches and BLW in infancy.

**Trial Registration:**

Australian New Zealand Clinical Trials Registry ACTRN12620000459921; http://www.anzctr.org.au/Trial/Registration/TrialReview.aspx?id=379436.

**International Registered Report Identifier (IRRID):**

DERR1-10.2196/29048

## Introduction

### Background

The biggest dietary change in every human’s life is the change from consuming a 100% milk diet in the first months of life to consuming a diet that is broadly the same as that of the rest of the family by the first birthday. This change is large. In fact, an infant following the current infant feeding guidelines globally [1-4] would have breast milk (or infant formula) as the sole source of nutrition until around 4 to 6 months of age. The first “complementary” foods would then be introduced one at a time in 1 to 2 teaspoon serves of “thin smooth purée,” followed by a progression in food texture from puréed to mashed to chopped, and by around 1 year of age, family foods are consumed. While many countries have reasonably up-to-date information on the nutrient intake of infants [5-7], there is no such comparable data in New Zealand infants. Additionally, no country has specific information yet on the impact of the revolution in infant feeding offered by the new phenomena of baby food “pouches” and “baby-led weaning” (BLW).

Baby food pouches are squeezable containers with a plastic spout described as a “mess-free and easy alternative for baby food on the go” [8]. They have immense appeal to parents for their convenience [9] and perceived superior safety and freshness over more traditional glass jars [9], and parental perceptions that they are healthy and enjoyed by the baby. This appeal is demonstrated by an extraordinary market share. A recent analysis of 24 major brands of infant and toddler foods in the United States (that represent more than 95% of market share) showed that 56% of the food products were packaged as squeeze pouches [10]. The market share continues to grow. Sales of baby food pouches in Europe have grown annually by 125% in Spain and 916% in the Ukraine [11]. Surprisingly, there appears to have been almost no direct research on the possible impact of this new technology on infant diet or health, despite several groups cautioning against the use, highlighting the urgent need for research on the safety, nutrition, and health impacts of pouches [12-14].

Although the foods offered in baby food pouches are broadly similar in content to those offered as baby foods in jars and cans, there has been some concern, albeit not universal [15], that the content of added sugar might be higher [10,13,14,16]. Certainly, cereal products in pouches are a poor source of iron and should not be used to replace iron-fortified infant cereals [15]. Regardless of nutrient content, the delivery method itself has the potential to markedly change infant nutrition for several reasons. Anecdotal reports suggest food products are being consumed straight from the pouch, unsupervised, and “on the go,” so they are outside usual eating contexts. This could have a number of important impacts on infant health and development.

First, these smooth highly processed products with multiple blended ingredients bear little resemblance to intact fruits and vegetables, and they are marketed well beyond the early weeks of complementary feeding when it might be argued a “super smooth” product is appropriate (eg, many are marketed for infants 8 months plus). This raises a number of questions. Do these products increase energy intake because they are so easy to eat (smooth consistency and do not require chewing; an entire 120-g serve can be accessed merely by squeezing the soft pouch and sucking)? Conversely, does the ease of consumption lead to displacement of other more nutrient-rich foods, such as breast milk (or infant formula), from the diet (eating “on the go” is unlikely to be consistent with New Zealand Ministry of Health recommendations that until 8 months of age infants are offered solid foods after milk to avoid displacing nutrient-rich milk [2])? Certainly, the structure of baby food pouches is described as “facilitating rapid passage of solid foods” [12], which suggests overeating is possible.

Second, if these pouches are indeed being consumed “on the go” by infants, what implications does this have for learning about food and eating? For example, do infants who feed themselves by sucking a purée out of a pouch while “on the go” have the same relationship with food as that for infants who sit and eat with their family? The description of pouches “promoting self-feeding and independence” [11] may suggest not. Positive reciprocal interaction during feeding or “responsive feeding” (ie, the caregiver and child respond appropriately to one another’s cues) may support the infant’s innate ability to respond to hunger and satiety cues [17]. If pouch use promotes “self-feeding and independence,” does frequent pouch use result in a lack of interaction during feeding? Moreover, is there any association between regular pouch use and the infant’s satiety responsiveness (ie, ability to cease eating once full)? Furthermore, parents are able to model healthy eating behaviors when the infant takes part in shared eating occasions, and this is associated with infants being less “food responsive” (ie, less likely to eat just because food is available) [18]. Beyond this, family routines and rituals around shared meals provide “a predictable structure that guides behavior and an emotional climate that supports early development” [19]. If feeding interactions and modeling influence the development of eating behaviors, then regularly eating from a pouch while “on the go” rather than in a shared eating setting may have important impacts on the development of infant eating behaviors.

Third, what is the impact of prolonged exposure to these often sweet and acidic (and therefore presumably cariogenic) foods on erupting teeth? There is a strong relationship between the frequency of cariogenic food intake and childhood caries [20]. Children who experience caries as infants or toddlers (ie, early childhood caries) have a much greater risk of developing caries in their permanent teeth [21], with children who have high exposure to sugars during infancy having a much greater risk of dental caries at 3 years than children who have less exposure to sugars in infancy [22]. Frequent consumption of sweet and acidic foods in early infancy may be of particular concern because newly erupted teeth have immature enamel and are more likely to develop caries [21]. Dental caries can have immediate and ongoing impacts on child health and quality of life, including reduced weight gain if food consumption is impacted, pain and discomfort, altered sleeping habits, and in the worst cases, hospitalization. Concerns have been raised about the possible impact of baby food pouches on pediatric dental health [12,23]. Certainly, advice to limit cariogenic foods to meal times [24] is not being followed if fruit- or cereal-containing pouches are used for snacks while “on the go” and therefore between meals.

While the use of food pouches is starting to be investigated internationally [25], there is no published research examining their relationship with infant nutrient intake, eating and feeding behaviors, growth, or dental health. The US Feeding Infants and Toddlers Study (FITS 2016) has collected but not yet reported data on the use of baby food pouches [25], but FITS 2016 did not collect data on nutritional status or health outcomes. Despite the lack of research on the possible impacts of pouch use, health professionals have expressed concerns [23,26-29]. A recent New York Times article [26] reports a spokeswoman for the American Academy of Pediatrics expressing concerns that pouch use may lead to children “overriding their body’s own cues for hunger and fullness” and recommending families should have established times for meals rather than “pouching the calories throughout the day.” In response to the article, a pediatric occupational therapist describes behaviors she has observed as follows: “Pouches appear to solve so many problems: kids who make a mess, kids who refuse fruits and veggies, kids who refuse to touch food or use a utensil, kids who won't sit still through a meal… I see toddlers waltzing through homes every day, sucking on pouches to start, end, or replace a meal” [26].

These are just anecdotal reports, but they underline the urgent need to determine the effect of pouches on infant nutrition and health. Interestingly, given the lack of research in this area, some health professionals in Germany [27] and the United Kingdom [23] have already gone so far as to recommend against the use of baby food pouches.

The second recent phenomenon in infant feeding is the popular adoption of BLW, an alternative approach to introducing solids to infants. In BLW, infants feed themselves all their foods from the start of the “complementary feeding” period. This means no spoon feeding by a parent, and only “finger foods” are offered [30]. BLW differs considerably from the more traditional approach espoused by infant feeding guidelines in many countries [1-4], in which the infant *gradually* learns how to eat solid foods safely by eating foods with progressively increasing textures from puréed to mashed to chopped to whole. We currently do not know how pervasive BLW is, although a recent New Zealand study [31] suggests more than half of families have tried it, with approximately 30% following it regularly. However, considerable concern has been expressed by health professionals about the potential increased risks of iron deficiency, growth faltering, and choking with BLW [32,33]. The limited international research base would suggest these concerns may be justified. We have shown previously in a small sample of infants aged 6 to 8 months that those following BLW had a much lower iron intake than traditionally fed infants [34], which is an issue given that iron is already a nutrient of concern in infants and toddlers, both in New Zealand [35] and internationally [36]. In order to truly know whether concern about low iron intake in infants following BLW is justified, the biochemical iron status of infants must be determined as iron intake is a notoriously poor indicator of iron status [37]. Only two small studies appear to have examined intake of other nutrients in infants following BLW, suggesting that intakes of zinc and vitamin B12 may be lower [34], and intakes of total fat, saturated fat [34], and sodium [38] may be higher than those for traditional spoon feeders. These important differences require clarification in a much larger sample. Proponents of BLW argue that infants are able to feed themselves sufficient food from 6 months of age and that allowing children to have control over their own eating promotes a greater ability to regulate their own appetite appropriately. Whether this is indeed true or translates into differences in growth rates is uncertain given that the few existing studies [39,40] have used parental reports, which can be inaccurate [41], particularly in infants who are growing so rapidly. Lastly, foods, such as raw apple, raw carrot, and grapes are some of the most commonly seen foods in videos promoting BLW, despite the substantial choking risk they pose to young children [42]. Whether choking rates differ in BLW versus more traditional solid feeding is not clear. Only one large study internationally has specifically investigated choking rates in infants following BLW rather than traditional spoon feeding (TSF) [43]. This study suggested that choking rates may in fact be lower for infants who consume finger foods regularly, but it recruited the BLW participants from BLW websites rather than the general population. This may have biased the results as choking and gagging are common topics discussed on BLW websites (particularly the importance of not mistaking gagging for choking), and this may have influenced the reporting of choking rates in the BLW infants. Given the substantial number of parents following this approach with their infants and BLW’s widespread online presence (7,690,000 results on Google; December 24, 2020), it is critically important to determine the health risks of BLW so that health professionals and policy makers can provide families with evidence-based advice on how to feed their infants safely.

Infant milk (breastmilk or infant formula) is a substantial component of the diet for infants during the complementary feeding period, providing more than half of their energy intake at 7 months of age [44]. While it is straightforward to estimate intake of infant formula for those who consume it, researchers currently have to use a “one size fits all” approach to estimate breast milk intake, either using a single volume for all breastfed infants of a particular age [45] or excluding breastfed infants from dietary analyses [46]. Neither approach is ideal because breast milk intake varies considerably between mother-infant pairs [47] and because breastfed infants do not necessarily have the same food intake or socioeconomic background as formula fed infants. The conventional method used to measure breast milk intake is to weigh the infant before and after every feed. However, this “test-weighing” technique is time consuming and may disturb usual feeding patterns. In contrast, the stable isotope deuterium oxide technique requires the mother to consume a small amount of the stable isotope (deuterium oxide) in water, and the amount of this marker transferred to the infant (ie, via the breast milk the infant consumes) is then measured by collecting saliva samples from the mother and infant over the following fortnight [48-50]. The normal feeding pattern is not disturbed, and the total volume of breast milk consumed by the infant over the fortnight can be accurately assessed. The use of this technique will allow the First Foods New Zealand (FFNZ) study to collect data that will enable more accurate estimates of nutrient intake in this age group and allow us to generate predictive models that use infant and diet characteristics that are routinely measured to estimate breast milk volumes. Such models would be invaluable for estimating total nutrient intake for breastfeeding infants (69% of infants at 4-8 months in New Zealand [51]) in future studies.

The FFNZ study will determine the iron status, growth, food and nutrient intakes, breast milk intake, eating and feeding behaviors, dental health, oral motor skills, and choking risk of New Zealand infants, with a particular focus on the use of baby food pouches and BLW.

### Primary Objective

In infants aged 7.0 to 9.9 months, we will determine whether iron status and BMI z-score differ according to the extent of food pouch use and complementary feeding approach (BLW compared with TSF).

### Secondary Objectives

In infants aged 7.0 to 9.9 months, we will estimate the following: (1) Nutrient intake, nutrient adequacy, and foods of cultural importance in New Zealand infants and in infants fed using baby food pouches regularly or those following BLW; (2) Breast milk intake in New Zealand infants and in infants fed using baby food pouches regularly or those following BLW; (3) Prevalence and nature of food pouch use; (4) Prevalence of BLW; (5) How eating behaviors (ability to eat with appetite, speed of eating, and picky eating) and feeding behaviors (parental responsiveness to infant hunger and satiety cues) differ according to the extent of food pouch use and complementary feeding approach (BLW compared with TSF); (6) How dental health differs according to the extent of food pouch use and complementary feeding approach (BLW compared with TSF); (7) How oral motor skills differ according to the extent of food pouch use and complementary feeding approach (BLW compared with TSF); and (8) How the risk of choking differs according to the extent of food pouch use and complementary feeding approach (BLW compared with TSF).

## Methods

### Design

The FFNZ study is an observational cross-sectional study of food and health in infants aged 7.0 to 9.9 months. The study will compare infants using baby food pouches with those not using these pouches, and those following BLW with those following TSF, while collecting data on nutrient intake and nutritional status in this age group in general. The age range has been chosen because it is close enough to when complementary feeding starts (usually 4-6 months of age) that we can expect to see large variations in both baby food pouch use and BLW rates, while also giving enough time from the start of complementary feeding for eating patterns to have had an impact on iron status and growth. A narrow age range has specifically been chosen because diet changes rapidly in infancy. Observational study designs are appropriate for identifying associations between behaviors as they are carried out in the “real world.” While a randomized controlled trial is required to determine causality, it is not ethical to randomize infants to follow BLW or pouch use because that would require randomization of participants to eating patterns that health professionals have concerns about [23,26-28,32,33]. Instead, we will use propensity score matching [52], which is able to remove some of the bias caused by differences in demographics between groups so that the estimates of the impact of pouch use or BLW on infant diet and health will more closely represent a potential causal effect [53].

### Participants and Recruitment

In total, 625 parents/guardians who have an infant less than 9.9 months of age will be recruited from two regions of New Zealand (Dunedin and Auckland) to participate in the study when their infant is aged 7.0 to 9.9 months. Recruitment will occur by advertisement and word of mouth and will target all infants rather than those adopting BLW, TSF, or food pouch use. We aim to recruit a sample that is broadly representative of the ethnicity and socioeconomic status of New Zealand children. It is not feasible to recruit a truly representative sample using typical methods, such as electoral roll and door knocking, because they would identify very few infants in the narrow age band that is necessary for this study (because diet changes so rapidly in infancy). We will, however, collect data from a diverse range of ethnic and socioeconomic groups by (1) engaging with Māori and Pasifika community health organizations to assist with recruitment, (2) targeting recruitment in suburbs with a high proportion of Māori, Pasifika, and Asian populations, and (3) having research team members who have experience working with or who culturally identify with Māori, Pasifika, and Asian communities. We will also statistically weight the estimates to account for demographic disparities if appropriate. The study has ethical approval from the Health and Disability Ethics Committees New Zealand (19/STH/151), and written informed consent will be obtained at the first appointment. The study is registered with the Australian New Zealand Clinical Trials Registry (registration number: ACTRN12620000459921).

### Sample Size

Our sample size calculation is based on comparing the BMI z-score and plasma ferritin concentration in infants following BLW and TSF, as there are currently no data internationally on these measures in pouch users. Our recent studies suggest that 29% of infants will meet the definition of BLW [31] and 70% of enrolled infants will provide a blood sample [54]. A recruitment size of 625 would therefore enable us to collect complete data from 125 BLW and 312 TSF infants, which would be sufficient to detect a difference of 0.3 for the BMI z-score and a 5-μg/L lower plasma ferritin concentration in the BLW group assuming a mean of 29 μg/L in the TSF group [54], both with 80% power and α of .05. As outlined above, we expect pouch use to be very common, based on the 70% market share they have, but we do not have the data needed to calculate the sample size required to detect differences in health outcomes with pouch use. With a sample size of 625, however, we will be able to estimate the prevalence of frequent pouch use to a 95% precision level of at least ±4%.

### Data Collection

#### Overview

Participation in the study will involve three (participants in the main study) or five (participants in a consecutive subsample of breastfed infants) contacts over 2 weeks following recruitment. For the main study (n=625), the first main appointment will generally be held in the participant’s home and involve a 24-hour diet recall, completion of two questionnaires, and anthropometric measurements of the child. The second main appointment will generally take place at university research rooms, and involve a second 24-hour diet recall and photography of the infant’s teeth to assess dental health. A third main appointment will take place at our university rooms (Dunedin) or a local blood testing facility (Auckland) to collect a blood sample to measure the iron status. Finally, a self-administered questionnaire will be completed by the participants in their homes. For the subsample (n=150) involved in the measurement of breast milk intake, a stable isotope will be given at the first main appointment, with three additional saliva samples collected over the ensuing fortnight (the third sample being collected at the second main appointment).

#### Demographic Data

At the initial appointment, ethnicity, maternal education, maternal work status, household deprivation (New Zealand deprivation index 18 [55]), household food security [56], and childcare use will be collected by questionnaires, using the New Zealand census questions where relevant. These data will be used to describe the sample and minimize bias.

#### Measuring Baby Food Pouch Use

This is related to primary objective 1 and secondary objectives 1, 2, 3, 5, 6, 7, and 8. We will measure the frequency of pouch use in the past month by a questionnaire. We intend to define infants as being frequent baby food pouch users if their parents state that they are currently being given food from a food pouch “5 to 6 times a week,” “once a day,” or “more than once a day,” although this may need to be modified when the distribution of intakes is determined (there are currently no published data on the frequency of baby food pouch use to base this cutoff on). We will collect data on the frequency of pouch use, use of “ready-to-eat” pouches versus “home-filled” pouches, extent to which the infant feeds directly from the pouch rather than being fed by spoon, types of foods given in “pouches,” contribution of pouch foods to total intake of solid foods, proportion of the “pouch” consumed on a typical eating occasion, duration of a typical eating occasion, physical situations in which pouches are used, proximity of an adult when the pouch is being used, reasons for using pouches rather than other methods of food delivery, and anything not liked about using baby food pouches. Key pouch questions will be asked referring to when the infant first started eating solids, when the infant was around 6 months of age, and “now.”

#### Measuring BLW

This is related to primary objective 1 and secondary objectives 1, 2, 4, 5, 6, 7, and 8. Parents will be asked to describe the way their infants were fed when they first started eating solids, when they were around 6 months of age, and “now,” using five answer options. Parents who choose “spoon fed by an adult” or “mostly spoon fed by an adult, some baby feeding themselves” will be classified as TSF. Parents who select “about half spoon feeding by an adult and half baby feeding themselves” will be classified as partial BLW [31]. Those who report “mostly baby feeding themselves, some adult spoon feeding” or “baby feeding themselves” will be assigned to full BLW [57,58]. As there is no validated definition of BLW, these definitions have been designed to capture the major point of difference between BLW and TSF, while allowing occasional adult spoon feeding.

#### Iron Status

This is related to primary objective 1. A nonfasting venipuncture blood sample will be collected at the third main appointment (3-mL EDTA anticoagulated vacutainer blood collection tube; Becton Dickinson and Company) to determine the plasma ferritin concentration and iron status defined using the body iron concentration (calculated using plasma ferritin and soluble transferrin receptor concentrations [36]) and hemoglobin concentration (from a complete blood count). The iron status categories are defined in Table 1 [54].

**Table 1:** Iron status categories.

Category	Body iron value	Hemoglobin value	Plasma ferritin value
Iron sufficient	≥0 mg/kg	≥105 g/L	≥15 μg/L
Iron depleted	≥0 mg/kg	≥105 g/L	<15 μg/L
Early functional iron deficiency	<0 mg/kg	≥105 g/L	N/A^a^
Iron deficiency anemia	<0 mg/kg	<105 g/L	N/A

^a^N/A: not applicable.

As ferritin is an acute phase reactant and can be artificially elevated by inflammation, we will also analyze two inflammatory markers (C-reactive protein and α-1-acid glycoprotein) as recommended by the World Health Organization (WHO) [59]. This will enable us to use the BRINDA (Biomarkers Reflecting Inflammation and Nutritional Determinants of Anemia) statistical method to adjust for inflammation on a continuous scale, consistent with the assumption that higher concentrations of inflammatory markers will be associated with a greater effect on plasma ferritin concentration [60].

Participants will be given verbal instructions on how to apply a local anesthetic (Ametop gel; Perstorp Pharma) and will be given access to an instruction video. The gel is to be applied to the insides of both of the infant’s elbows (this enables phlebotomy to be attempted on the second arm if necessary) and covered with an occlusive dressing at least 1 hour (no more than 4-6 hours) before the blood test appointment. It is removed after 30 to 45 minutes. The blood sample will be taken by a pediatric phlebotomist. The research team has extensive experience overseeing research projects involving the collection of venipuncture blood samples from infants and toddlers [54,60,61], with 70% [54] to 92% [61] of participants providing samples.

Commercial laboratories (Southern Community Laboratories, Dunedin, New Zealand and Labtests NZ, Auckland, New Zealand) will determine complete blood count (requires fresh blood) and plasma ferritin, so that the iron status can be immediately communicated to the infant’s general practitioner if the infant is identified as having anemia. The remaining plasma will be frozen at −80°C for batch analysis of soluble transferrin receptor, C-reactive protein, and α-1-acid glycoprotein concentrations at the University of Otago Department of Human Nutrition Laboratory at the end of the study [54].

#### Anthropometry

This is related to primary objective 1. Infant weight will be measured at the initial appointment on an electronic scale (model 354; Seca) and length will be measured on a 99-cm measuring mat (model SE210; Seca) in duplicate following WHO protocols [62]. BMI (weight in kg divided by height in meters squared) will be calculated, and BMI-for-age z-scores will be determined using WHO reference data [63]. We will also request consent from parents to collect information on BMI from the B4 School Check [64] when the children are 4 years of age. This will enable us to look at the effects of pouches and BLW on growth longitudinally.

#### Infant Diet

This is related to secondary objective 1. Information on infant nutrient intake and adequacy, food group intake, dietary patterns, and culturally important foods will be obtained using interviewer-administered multiple pass 24-hour recalls collected at the first and second appointments. The two 24-hour recalls take place on different days of the week to capture variation in intake between days. Collecting two 24-hour recalls will enable us to calculate “usual intake” using the multiple source method (MSM) for estimating usual dietary intake of individuals [65]. Photograph prompts will be used to assist recall of foods eaten. Participants will be asked to photograph (using their own smartphone or a camera provided) the eating surface (eg, plate, high chair surface, and table) at the start of all meals and snacks from midnight to midnight on the day before the appointment. The quality of the photographs will not be important as long as they are clear enough to remind the parent what the child ate. Diet recalls will be analyzed with FoodWorks (version 10, Xyris Software) using the New Zealand Food Composition database FOODfiles 2018 Version 01 [66]. Nutrient information for commercial infant foods and milk will be determined by generating recipes using the ingredients lists on food products modified to match the nutrient information panel on the packet so that the contribution of nutrients that do not appear on the nutrient information panel can be included [15]. Information on supplement use will be collected by a questionnaire.

Particular focus will be on free sugars and added sugars given a recent small study [45] suggested that even by 7 months of age, 12% of New Zealand infants may already be consuming free sugars at levels that are above the WHO recommendation [67]. Parents are discouraged from adding sugar to infants’ diets because it is unnecessary and may increase liking of sweet foods [2]. In addition, the WHO recommends that free sugars should be <5% of energy intake due to the dose-response relationship between free sugar intake and dental caries (even in populations with water fluoridation) [67]. Data on free sugars and added sugars are available in the New Zealand food composition database [66,68].

Questionnaire and 24-hour recall data will also be used to determine the extent to which key indicators of diet quality are being met, as guided by the New Zealand Ministry of Health Eating and Activity Guidelines for New Zealand Infants and Toddlers [2].

#### Breast Milk Intake

This is related to secondary objectives 1 and 2. We will obtain accurate data on the amount of breast milk infants consume using the stable isotope (deuterium oxide) “dose-to-mother” technique [48-50] in a consecutive sample of 150 breastfeeding mother-infant dyads. This will enable us to measure intake to ±34 mL/day (ie, 5% of expected total intake [47]) at a 95% precision level. The isotope will be administered to the mother orally at the initial appointment (after collection of the baseline saliva sample), and saliva samples will be collected from the mother and infant at three further appointments to measure the disappearance of the deuterium from the mother and appearance in the infant. Baseline and three postdose sampling points (days 2-3, 7-9, and 13-14) are required to achieve adequate accuracy and precision of human milk intake. Height/length and weight will be measured at baseline, with weight measured again at the final appointment for both mother and infant, so that breast milk intake can be calculated. Breast milk intake data will be used along with questionnaire and recall data to generate predictive equations of breast milk intake so that intake can be estimated for participants who did not have breast milk intake measured.

#### Eating Behaviors

This is related to secondary objective 5. Eating behaviors will be assessed using the following four subscales from the Children’s Eating Behavior Questionnaire (CEBQ) [69]: “satiety responsiveness” (eating appropriately in response to appetite), “food responsiveness,” “food enjoyment” (eating in response to environmental food cues rather than hunger), and “slowness in eating.” Although a Baby Eating Behavior Questionnaire has been developed [70], it is designed for infants who are exclusively milk fed, so it would not capture complementary feeding. Food fussiness will be measured using five items in the “picky eating” subscale of the Toddler-Parent Mealtime Behavior Questionnaire [71]. We have demonstrated the internal consistency and reliability of these scales in New Zealand infants at 12 months of age, with Cronbach α ranging from .83 to .90 [58]. Participants will also be asked whether they feed their infants any foods of particular cultural relevance. Feeding behaviors will be determined by observing how infants eat and how parents react in response to hunger and satiety cues by videotaping one evening meal (at which solid foods are offered) in each infant recruited in the Dunedin cohort (n is approximately 300). Participants will be issued a GoPro wide-angle video camera (Hero 2018; GoPro Inc) and tripod at their first appointment and asked to video record the main meal on the day for which the second 24-hour recall will be obtained. The camera will take a continuous video from approximately 10 minutes before the infant first joins the meal to when they leave it. Videos will be coded using the Responsiveness to Child Feeding Cues Scale [72].

#### Dental Health

This is related to secondary objective 6. Photographs of the infant’s upper and lower teeth will be taken by trained interviewers using a dedicated study Oppo Reno2 Z (Oppo) mobile phone with a small portable Smile Light MDP lighting source specifically designed for taking dental pictures [73]. These images will be examined by a single registered dental practitioner, with blinded evaluation of a subset by another examiner, using validated indices for caries and developmental defects of enamel, which have a positive correlation with dental caries [74].

#### Oral Motor Skill Development

This is related to secondary objective 7. The questionnaires administered at the final appointment include the validated Child Oral Motor Proficiency Scale (ChOMPS) to identify oral motor and eating skill delay [75,76] and the Pediatric Eating Assessment Tool (PediEAT) to measure behaviors that characterize symptoms of feeding difficulties [77,78]. Both questionnaires have age-based reference values for infants and rely on parent reporting [79,80].

#### Choking

This is related to secondary objective 8. The questionnaire administered at the initial appointment will include questions on choking since birth. We developed these retrospective questions for previous work in this age group and have demonstrated that they provide data that are comparable to choking data collected prospectively using a daily choking calendar [81].

### Statistical Analysis

We expect that there will be crossover between BLW/TSF status and pouch use. We will be able to explore whether mean differences in energy and nutrient intake, as well as other measures, between pouch users and nonusers are different for those who use BLW and those who do not. These results will be stratified by BLW/TSF status, and estimated differences will be compared.

Regression models will be used to determine differences between groups. Propensity score matching will be undertaken to reduce the bias caused by differences in demographics between the groups (eg, maternal education and ethnicity), infant age (to the nearest week), and sex. Propensity score matching is not like traditional paired matching, where each individual is matched to another individual in the other group according to covariates. Instead, propensity score matching uses a participant’s propensity score (found using covariates) and estimates what their outcome (eg, energy intake) would have been if they were in the other of two dichotomous groups. By using this method, we expect the estimates will more closely represent a potential causal effect [53]. Another advantage is that it allows data from the whole sample to be used, unlike a traditional matched analysis in which only matched pairs are analyzed.

Estimates of BLW, frequent pouch use, nutrient intake, status, and adequacy will be calculated for the whole sample along with 95% CIs. If the sample is not demographically representative of the wider population, statistical weighting of these estimates will be undertaken using the survey command in Stata (StataCorp).

Nutrient intake will be determined using 24-hour recall data adjusted to provide estimates of usual intake (using the MSM method [65]). Adequacy of intake will be determined as follows: for zinc (for which an estimated average requirement [EAR] is available for New Zealand and Australia [82]), the EAR cut point method will be used; for iron (for which an EAR and tables of probabilities of inadequate intakes are available for the United States of America and Canada [83]), the full probability approach will be used [83]; for other nutrients (only an adequate intake [AI] is available for this age group in New Zealand and Australia [82]), mean group intake above the AI will be considered to indicate adequacy, but a conclusion as to inadequacy will not be possible if mean group intake is below the AI [84]. The BRINDA method [60] will be used to adjust plasma ferritin, and therefore, body iron and iron status, for the impact of inflammation.

The best-fitting polynomials to predict breast milk intake will be estimated by regression models using fractional polynomial functions of variables, such as age, sex, body weight, and food and beverage intake (eg, kJ/day). This will result in equations that can be used to predict breast milk intake based on a variety of input variables. Ideally one based on data that can be collected in a single clinical appointment, and another that uses data requiring more extensive collection in a research or surveillance setting.

## Results

This observational study has full ethical approval from the Health and Disability Ethics Committees New Zealand (19/STH/151) and was funded in May 2019 by the Health Research Council (HRC) of New Zealand (grant 19/172). Data collection commenced in July 2020, and the first results are expected to be submitted for publication in 2022. Data collection will only take place while New Zealand is in Alert Levels 1 or 2 during the COVID-19 pandemic. The Otago and Auckland regions of New Zealand, where the data collection will take place, have been in Level 1 for all but 7 weeks in Otago (all at Level 2) and 11 weeks in Auckland (7 weeks at Level 2 and 4 weeks at Level 3) since July 2020, as overall case numbers in New Zealand remain extremely low (<2000 in a population of more than 5 million). As daily life is essentially normal in Level 1 with the exception of closed international borders and Level 2 just requires some physical distancing and appropriate hygiene recommendations, we feel confident that the pandemic will have relatively little effect on our data.

## Discussion

This large observational study will provide much needed data on nutrient intake (including breast milk intake) and nutritional status (specifically iron status, growth, and dental health) in a large diverse sample of New Zealand infants. However, our data will also have considerable international appeal given the lack of research assessing the implications for nutritional intake and health for those infants who obtain a large proportion of their food via baby food pouches. Similarly, determining how iron status, growth, nutrient intake, and choking risk may differ in infants following BLW compared with TSF is urgently warranted given the widespread interest in this alternative approach to complementary feeding worldwide.

## Appendix

Multimedia Appendix 1 Peer-reviewer reports and grant approval from Health Research Council of New Zealand.

Multimedia Appendix 2 Ethics approval.

## Abbreviations

AI: adequate intake
BLW: baby-led weaning
BRINDA: Biomarkers Reflecting Inflammation and Nutritional Determinants of Anemia
EAR: estimated average requirement
FFNZ: First Foods New Zealand study
FITS: US Feeding Infants and Toddlers Study
MSM: multiple source method
TSF: traditional spoon feeding
WHO: World Health Organization
