# Supplementary material for: Nutritional Implications of Baby-Led Weaning and Baby Food Pouches as Novel Methods of Infant Feeding: Protocol for an Observational Study
Source: JMIR Res Protoc. 2021 Apr 21;10(4):e29048. doi: 10.2196/29048 (PMC8100878; doi:10.2196/29048)
Supplement: Multimedia Appendix 1 [file resprot_v10i4e29048_app1.pdf]

Applicant peer review report

Reviewer # 27

Proposal details

Title	Novel methods of infant feeding in New Zealand - cause for concern or optimism?
First named investigator	Associate Professor Anne-Louise Heath (University of Otago)

Rationale for research — Score: 7

This research is well-conceived, timely and addresses potentially significant health issues facing infants and their families. Knowledge generated from this research will have broader impacts on the international context, relevant to but also beyond NZ. Most importantly, this research and action plan thought through by the team has the potential to better inform the public regarding infant feeding decisions. The research objectives are simple and clear, each addressing a profound knowledge gap. I commend the research team for the originality of this idea. The project is also culturally appropriate for the Maori population, and I believe objectives related to the Maori population will yield interesting and much needed results.

Design and methods — Score: 6

The cross-sectional study design is feasible; the researchers have provided a clear justification for the cross-sectional design. I believe that, with the number and diversity of researchers on the team, the project will be completed within the proposed timeframe. The researchers propose a multi-site study with clear actions to be undertaken at both sites (Dunedin and Auckland), which I believe will adequately capture the intended audience. The researchers endeavor to collect rigorous biological and dietary measures (i.e., iron status, dietary recall and breastmilk consumption) and will attempt to move the field forward using some of these novel methods (relevant to the NZ and greater global population). I suggest that the researchers have a clearer idea regarding the data coding and analysis for the timelapse photo observation. While there was sufficient justification for choosing to analyse photos over video, I am concerned that the context of the feeding event may be missed. Nevertheless, this depend on what is being coded, and therefore was insufficient information for me to make a decision if this method was appropriate. The research team includes a biostatistician, who will undertake the analysis.

Research impact — Score: 7

The research team asks an important, timely question in feeding methods during complementary feeding. The details the research team are collecting will contribute significantly to not only the research field, but to the health of the NZ population - and possibly, globally.

Team: research outcomes — Score: 7

The team has a proven record in completing high-impact and relevant research to this project. I believe the team has demonstrated a good, multidisciplinary mix of professionals and skills. The team is a capable of carrying out the project successfully.

General comments
