# Supplementary material for: Nutritional Implications of Baby-Led Weaning and Baby Food Pouches as Novel Methods of Infant Feeding: Protocol for an Observational Study
Source: JMIR Res Protoc. 2021 Apr 21;10(4):e29048. doi: 10.2196/29048 (PMC8100878; doi:10.2196/29048)
Supplement: Multimedia Appendix 2 [file resprot_v10i4e29048_app2.pdf]

Applicant peer review report

Health Research Council of New Zealand

Reviewer # 31

Proposal details

Title	Novel methods of infant feeding in New Zealand - cause for concern or optimism?
First named investigator	Associate Professor Anne-Louise Heath (University of Otago)

Rationale for research — Score: 6

1 Yes. Diet during infancy is critical for good nutrition

2. Yes. There is limited data on the dietary intakes of NZ children and there is evidence that weaning practices and weaning foods have changed dramatically in recent years

3. Yes. Opportunity to provide clear recommendations to health professionals and parents and identified feasible pathway

4. Yes. The study will be able to address specific research questions relevant to Maori

The rationale is well argued although reference to acidic foods being carcinogenic seems peripheral to the caries issue and consequently a little inflammatory. More information on the origins of BLW would strengthen the rationale (is it industry driven)?

Design and methods — Score: 5

Design

Well justified noting the following potential weaknesses

* Targeting all infants is important for reducing bias but how can we be sure the study will yield sufficient numbers of mothers who frequently use pouches or BLW or sufficient variability in socio-demographics (particularly ethnicity) to answer the research questions? No strategies are mentioned for recruiting or retaining Maori, Pacific or Asian participants. Also, will the researchers try and achieve quotas as a way of overcoming low response, particularly for the sub-sample who will be asked to consume deuterium dioxide? This seems like a better strategy than weighting and information was not provided on what the data would be weighted to?

*BLW hasn't been defined or measured before so it is unclear if the variable will capture what the researchers are after and what variation there will be across the categories. For example, Is baby led weaning the same as baby feeding themselves? I can see the degree of involvement with an infant during eating creating a distinction.

* Is pouch use BLW? How will the independent impacts of pouch use and BLW be determined?

Methods

*Appropriate data collection tools and methodology for anthropometrics, eating behaviours, dietary intakes and biochemistry. Would like to have learned more from the pilot about how complete data recording was and behaviour modification in response to camera presence. *Has the process of taking photos of infants teeth been piloted? Also is the DDE suitable for use with such young children?

*How will the 4yo School Check Dental data be accessed?

*A process for ethical approval is not described

Research impact Score: 5

*A credible pathway for how research will be translated into benefits was described and the methods should deliver results with a reasonable level of robustness. It is easy to see for example how the results may influence guidance to mothers from Plunket or the Ministry of Health and the researchers have identified important pathways for informing food industry. However, because it is cross-sectional and the first study of its kind it would be overstating the impact to say it will directly influence policy

*I wonder if there is a bias against BLW in the research team (ie they have already conducted an RCT to overcome nutrition and choking issues associated with this eating pattern) that needs to be managed?

*While not the primary aim, the value of the study for gathering information on infant foods of cultural importance to Maori should not be underestimated. This information is clearly valued by Maori advisors to the project and has the potential to make intake24 a more culturally sensitive dietary collection tool and to stimulate further research on culturally appropriate infant feeding practices.

Team: research outcomes Score: 6

*Considerable track record (including 7 HRC grants) and expertise in the broad content areas and methodologies of the application, including the recruitment of women for a similar study.

*Strong contribution to those delivering Health Services in New Zealand

*Co-leading arrangement between NI Heath and Taylor should be articulated further to include time split and responsibilities.

*Total investigator FTE is 0.48

General comments

This is a well written grant that explores the understudied area of infant feeding practices. The named investigators are experienced in this area of research, have recognised the considerable knowledge gap and have proposed the collection of a wide range of date to fill it using a suitable methodology. Pathways for disseminating the finds and translating them into action have been articulated.

I had some some concerns about the feasibility of the grant based on an overall Named Investigator FTE of 0.48 and RA input of 1.0 FTE? These will diminish if the PhD students are also contributing substantial time to the data collection etc but this was not clear and funding for Isabel Carlisle seems to be missing from the budget
